# BitterSweetForest: A Random Forest Based Binary Classifier to Predict Bitterness and Sweetness of Chemical Compounds

**DOI:** 10.3389/fchem.2018.00093

**Published:** 2018-04-11

**Authors:** Priyanka Banerjee, Robert Preissner

**Affiliations:** Structural Bioinformatics Group, Institute for Physiology and ECRC, Charité – University Medicine Berlin, Berlin, Germany

**Keywords:** Random Forest, bitter prediction, sweetness prediction, fingerprints, KNIME workflow, taste prediction

## Abstract

Taste of a chemical compound present in food stimulates us to take in nutrients and avoid poisons. However, the perception of taste greatly depends on the genetic as well as evolutionary perspectives. The aim of this work was the development and validation of a machine learning model based on molecular fingerprints to discriminate between sweet and bitter taste of molecules. BitterSweetForest is the first open access model based on KNIME workflow that provides platform for prediction of bitter and sweet taste of chemical compounds using molecular fingerprints and Random Forest based classifier. The constructed model yielded an accuracy of 95% and an AUC of 0.98 in cross-validation. In independent test set, BitterSweetForest achieved an accuracy of 96% and an AUC of 0.98 for bitter and sweet taste prediction. The constructed model was further applied to predict the bitter and sweet taste of natural compounds, approved drugs as well as on an acute toxicity compound data set. BitterSweetForest suggests 70% of the natural product space, as bitter and 10% of the natural product space as sweet with confidence score of 0.60 and above. 77% of the approved drug set was predicted as bitter and 2% as sweet with a confidence score of 0.75 and above. Similarly, 75% of the total compounds from acute oral toxicity class were predicted only as bitter with a minimum confidence score of 0.75, revealing toxic compounds are mostly bitter. Furthermore, we applied a Bayesian based feature analysis method to discriminate the most occurring chemical features between sweet and bitter compounds using the feature space of a circular fingerprint.

## Introduction

Taste plays an integral role in determining the quality of food irrespective of their nutritive value. In human, taste can additionally contribute to the overall pleasure and satisfaction of a food or drink. Within the five basic tastes, sweet taste enriches our capacity to identify the energy rich-food (Morrison, 1979). Sweeteners are the compounds that interact with the sweet receptor and evoke a characteristic response and enhance the perception of sweet taste (DuBois and Prakash, 2012). Sweetness has long been known as a property possessed by many substances other than sugar, although their use as sweeteners have largely been confined to pharmacy or precluded by toxicity (Lindseth et al., 2014). The first commercially developed sweetener dates from the discovery of saccharin in 1879 by C. Fahlberg and I. Remsem (Dwaine, 1978). Artificial sweeteners are increasingly used as an alternative to sugar (Bellisle, 2015). Advancing incidence of obesity, diabetic and metabolic syndrome, coupled with heightened consumer awareness, has led to a steady paradigm shift toward the use of low calorie artificial sweeteners (Sharma et al., 2016). The relative global market share of the major sweeteners includes aspartame (29%), sucralose (28%), cyclamate (16%), saccharin (13%), stevia (9%), acesulfame-K (5%), and neotame (1%) (Tandel, 2011; Schiffman, 2012). It has been found that over last three decades, the percentage of people that use products containing sweeteners in the United States (Tandel, 2011), has more than doubled (Schiffman, 2012). On the other hand, bitter taste of a food is evolutionary linked to guarding against consumption of poisons (Reed and Knaapila, 2010). Bitterness is exhibited mostly by alkaloids (Levit et al., 2014). However, not all bitter compounds are toxic; many dietary phytonutrients commonly found in fruits and vegetables, as well as many clinical drugs and herbal based medicines elicit characteristic response of bitter taste (Bahia et al., 2017). The bitter tasting drugs are a major concern of compliance for children (Levit et al., 2014). Sensory tasting of drug candidates by human is not a trivial matter, since it requires ethical approval, which is achievable only after a thorough toxicological study (Levit et al., 2014; Bahia et al., 2017). Thus, efficient prediction of compounds sweetness as well as bitterness is not only a great interest of the nutrition industry and basic taste research but also for the drug discovery process. This is a space where the cheminformatic based *in silico* models can play a major role in supporting and advancing the research related to taste chemistry (Di Pizio and Niv, 2014). Even after the novel breakthrough in the structure determination of the G-protein-coupled receptors (GPCRs), the complete resolved structure of chemoreceptors is still not available (Di Pizio and Niv, 2014). Thus the computational models in general and ligand based computational models in particular, are essential support for the research in this area.

From the chemical point of view sweet and bitter compounds have some common chemical features, they do not generally dissociate or ionize in solution, on the other hand the compounds which cause the sour and salt tastes tends to ionize (Schiffman et al., 1995). Aside from this generalization, there are certain chemical features which are overrepresented in bitter molecules compared to sweet molecules and vice versa, which correspond to the extreme taste of the compounds (Schiffman et al., 1995). When attempts are made to relate sweetness or bitterness to the structure of chemical compounds, the most valuable sets of data arise when “relative” sweetness or bitterness scores are assigned to closely related compounds (Reed and Knaapila, 2010). It is a well-known fact that some changes in the chemical group in a sweet compound may change the sweet taste of that compound to either tasteless or bitter. For example, the sweet compound, saccharin, tastes bitter when a chloride or a methyl group is introduced in the meta position, while when the amino group is replaced by a methyl, ethyl, or bromoethyl radical results in the loss of the sweet taste, that is, the compound becomes tasteless (Schiffman et al., 1995).

Several computational approaches have been developed to predict either “sweetness” or “bitterness” of chemical (Huang et al., 2016; Rojas et al., 2017). Such as Quantitative structure-activity relationships (QSAR) based models are developed to build mathematical relationships between the chemical structures as defined by their molecular descriptors, and their respective properties (Rojas et al., 2017). Recently machine learning model based on physicochemical descriptors as well as commercially available adsorption, distribution, metabolism, and excretion (ADME/Tox) descriptors were developed to predict bitterness of compounds (Dagan-Wiener et al., 2017). Machine learning models using chemical based descriptors are widely accepted and used to predict different activities from molecular structures, such as biological activity (Lavecchia, 2015; Banerjee et al., 2016), different toxicity endpoints (Livingstone, 1994; Krewski et al., 2010). Many models for prediction of sweet and bitter taste are published in literatures, numerous commercial and web-server based models have also been available (Huang et al., 2016). However, an analysis of discriminating chemical features space between sweet and bitter molecules has not been reported yet.

In this paper, we have developed a Random Forest (RF) based model—BitterSweetForest for the computational prediction of sweet and bitter taste of chemical compounds using molecular fingerprints. In this study, we analyzed the relative frequency of the features overrepresented in the bitter and sweet compounds. The top features for each class (sweet/bitter) from the compound dataset were obtained from the calculation of the Bayesian probability for each feature represented in a compound fingerprint. The model was trained on a training set consisting of 961 compounds and evaluated with an independent test set of unseen 241 compounds. The constructed model yielded an accuracy of 95% and an AUC of 0.98 in cross-validation. In independent test set, BitterSweetForest achieved an accuracy of 96% and an AUC of 0.98 for both bitter and sweet taste prediction. Finally, the constructed model was used to predict the sweet/bitter taste of the compounds from SuperNatural II database (Banerjee et al., 2015), the approved drugs from DrugBank (Wishart et al., 2017) database and oral toxicity data from ProTox (Drwal et al., 2014).

## Materials and methods

### Data preparation

A total of 1,202 of chemical compounds consisting of both artificial and natural sweeteners (517 chemical structures) as well as bitter compounds (685 chemical structures) was extracted from SuperSweet (Ahmed et al., 2011) and BitterDB databases (Wiener et al., 2012). All the data structures were standardized using the Instant JChem software (version 6.2, Chemaxon) using the followings steps: water molecules were removed, molecules were aromatized, adjacent positive and negative charges transformed into double/triple bonds, and explicit hydrogen was added. InChIKeys were calculated using RDKit (http://www.rdkit.org) nodes in KNIME (Berthold et al., 2008) in order to identify and remove duplicates.

### Molecular representation

Structural features of the chemical compounds were represented by means of binary fingerprints. Binary fingerprints are widely used in molecular similarity searching methods and classification tasks. Four different types of fingerprints were used in the model, to evaluate individual performance of the fingerprints on the external set. They are Morgan fingerprint (2,048 bits), atom pair fingerprints (1,024 bits), torsion fingerprint (1,024 bits) and Morgan Feat fingerprints (2,048 bits). All fingerprints were calculated using RDKit node in KNIME (Berthold et al., 2008).

### Model construction

Random Forest algorithm is an ensemble learning approach, that constructs a large number of decisions trees, and outputs predictions that are collection of the votes of the individual trees. A subset of the training dataset is chosen to grow individual trees, with the remaining samples used to estimate the optimal fit. Trees are grown by splitting the training set (subset) at each node according to the value of the random variable sampled independently from a subset of variables. The number of trees in our RF model was set to 1,000, with greater value showing no further improvement. RF model was implemented using the Tree Ensemble Learner and Predictor nodes in KNIME (Berthold et al., 2008). The split criterion Gini index is used, which has previously been proven to be a good choice (Breiman, 2001). Additionally, a square root function was used for attribute sampling and different sets of attributes were chosen for all the trees. The total data was divided into 80% training data and 20% as validation set.

### Model performance

The total dataset consisted of 1,202 molecules, out of which 517 molecules were sweet and 685 molecules were bitter in taste. Data sets were randomly divided into 80% training set and 20% test set, ensuring the original distribution of the different classes (bitter and sweet). The training set included 961 molecules (416 sweet molecules and 545 bitter molecules) and the test set was comprised of the remaining 241 molecules (102 sweet molecules and 139 bitter molecules). The models using different fingerprints were trained on the training data and models parameters were tuned accordingly and then the models were evaluated on leave one-out cross validation (LOO) to avoid over fitting.

BitterSweetForest predicts new compounds (taste class) as continuous real-valued numbers in the range between 0 and 1, which describes the probability of the corresponding classes. However, the expected values recorded for each compound are binary (S = Sweet, B = Bitter). The quality of the model was evaluated for both the classes and cross-validation and external validation sets by means of sensitivity/recall, specificity, precision, accuracy, f-measure, Receiver Operating Curve- Area Under Curve (ROC-AUC), and Cohen's kappa (Gwet, 2008).

*Accuracy* describes how well the machine learning method correctly identifies the samples of the dataset.

$$Accuracy\;=\frac{\Sigma \text{ True positives}+\;\Sigma \text{ True Negatives}}{\Sigma \text{ Positives}+\;\Sigma \text{ Negatives}}$$

*Sensitivity* describes the true positive rate i.e., the numbers of positive compounds were correctly identified as positive.

$$Sensitivity=\;\frac{\Sigma \text{ True Positives }}{\Sigma \text{ True Positives}+\;\Sigma \text{ False Positives}}$$

*Specificity* is defined as the true negative rate i.e., the numbers of negative compounds were correctly identified as negative.

$$Specificity=\frac{\Sigma \text{ True Negatives}}{\Sigma \text{ True Negatives}+\;\Sigma \text{ False Positives}}$$

*Precision* is defined as the number of true positives divided by the number of true positives and false positives. It is also called the Positive Predicted Value (PPV).

$$Precision=\;\frac{\Sigma \text{ True Positives}}{\Sigma \text{ True Postitives}+\Sigma \text{ False Positives}}$$

*Recall* is defined as the number of true positives divided by the number of true positives and the number of false negatives, also called sensitivity or the true positive data.

*F-measure* is the weighted average of precision and recall.

$$F\text{-}measure=\;2\;*\;\frac{\text{Precision }*\text{ Recall}}{\text{Precision}+\text{Recall}}$$

*A receiver operating characteristic (ROC)-curve* is the plotting of the true positive rate against the false positive at various discrimination thresholds and is commonly used in binary classification. On the unit ROC space, a perfect prediction would yield an AUC of 1.0 and random results will be in points along with the diagonal with an AUC value of 0.5. The area under the curve (AUC) was used to measure the performance of the model both on cross-validation and external validation set (van Erkel and Pattynama, 1998).

*Cohen's kappa* is usually described as the amount of agreement correct by the agreement expected by chance. Cohen's Kappa is always less than or equal to 1. According to the scheme provided by Landis and Koch (Gwet, 2008) a value of < 0 indicates no agreement, and 1 as almost perfect.

## Results

In this study, we constructed a RF-based classifier: BitterSweetForest for the prediction of compounds tasting sweet or bitter. Initially, the model was trained based on four different fingerprints (Morgan, Morgan-feat, atom pair and torsion fingerprints), out of which the model based on Morgan fingerprint performed slightly better compared to the other three, as shown in Supplementary Table 1. The performance of the model was validated in leave-one-out cross validation and on an independent test set.

The BitterSweetForest for the prediction of sweet compounds has an accuracy of 0.95 and AUC of 0.98 on cross-validation and an accuracy of 96.69 and AUC of 0.98 shown in Table 1. The Cohen's kappa of the model is above 0.80 in both the sets, indicating the model as almost perfect. When compared to the published top performing models based on *k*NN (Rojas et al., 2017) and QSTR (Rojas et al., 2017) based methods, BitterSweetForest performs slightly better in terms of NER, Sensitivity and specificity both on training and test set as shown in **Table 3**.

**Table 1:** Cross-validation and external set validation results for sweet prediction.

	**Positives**	**Negatives**	**Accuracy (%)**	**ROC-AUC**	**Sensitivity**	**Specificity**	**Cohen's Kappa**	***F*-measure**
Cross-validation	416	545	95.00	0.98	0.90	0.97	0.82	0.94
External validation	102	139	96.69	0.98	0.91	0.97	0.92	0.92

Similarly, the BitterSweetForest for the prediction of bitter compounds has an accuracy of 0.95 and AUC of 0.97 on cross-validation and an accuracy of 96.69 and AUC of 0.98 as provided in Table 2. The Cohen's kappa of the model is above 0.80 in both the sets, indicating the model as almost perfect. When compared to the published top performing models, BitterSweetForest outperforms Bitter X (Huang et al., 2016) in terms of accuracy, achieving 7% more accuracy on training set and almost 5% more on test set, see **Table 4**. When compared to the BitterPredict (Dagan-Wiener et al., 2017), BitterSweetForest achieved 6% more in terms of sensitivity on training set and 20% more sensitivity on the test set as shown in **Table 4**. Thus, indicating that feature encoded molecular fingerprints can be used as optimal descriptors for the prediction of sweet and bitter taste of compounds. BitterSweetForest has achieved good performance in various parameters used in this study to evaluate the quality of the model and its prediction as shown in Tables 1, 2. Additionally, an external data set including sweet and tasteless compounds, as well as bitter and tasteless compounds was validated. The predicted performances of the model for both the classes were good with an AUC value of 0.85 and 0.90 respectively. This was done to make sure that the model is able to differentiate between sweet, bitter and tasteless compounds using the features. The model was not trained on tasteless compounds; this suggests that the features learned in the model are very specific to sweet and bitter compounds.

**Table 2:** Cross-validation and external set validation results for bitter prediction.

	**Positives**	**Negatives**	**Accuracy (%)**	**ROC-AUC**	**Sensitivity**	**Specificity**	**Cohen's Kappa**	***F*-measure**
Cross-validation	545	416	95.00	0.97	0.97	0.90	0.82	0.96
External validation	139	102	96.69	0.98	0.97	0.91	0.92	0.95

### Chemical features of sweet and bitter tasting compounds

Sweet and bitter taste properties are found in most classes of chemical compounds and a close relationship is found in many structural categories (Reed and Knaapila, 2010). A single compound can have both sweet and bitter features, and small structural modifications can result in change in the ratio of sweet and bitter taste intensities (Schiffman et al., 1995). On the other hand, some evidences suggest that sweet and bitter compounds have properties which are rather independent (Cardello, 1981). However, often most of the sweet compounds tend to taste bitter at different concentration e.g., aspartame, glucose, lacitol, malitol (Schiffman et al., 1995).

In order to understand which features, contribute most to the change in the expected class in the BitterSweetForest classifier, we analyzed the relative frequency of each features of the Morgan fingerprint in the respective sweet and bitter class.

One of the main purposes of this study was to analyze the important and frequent features in sweet and bitter compounds. The percentage of occurrences of each feature from Morgan fingerprint (2,048 bits) in sweet and bitter compounds was calculated. The relative frequency of important features for a class (e.g., sweet) were calculated taking not only the feature position and occurrence within the sweet class into account but also the relative feature frequency of that particular feature in the bitter class and vice versa. The average relative frequency for each class were calculated, a feature was only considered active for a class, if it's presence in one class is higher than the average relative frequency of that class as well as lower than the average relative frequency of the other class. The top features for each class were calculated using class-specific weighted bits/feature patterns in the fingerprints.

The Bayesian based Feature detection applied in the study, calculates the probability of any compound containing a feature (F) from the Morgan -feature space belongs to a specific class (e.g., Sweet or bitter), given that total number of the compounds containing the feature (F) and the number of compounds of feature (F) belong to that class. The dissimilarity (uncommon feature score between two classes) between the two features is calculated by the 1- Pearson correlation coefficient of their individual class specific scores (Bender et al., 2006).

**Figure 1:**
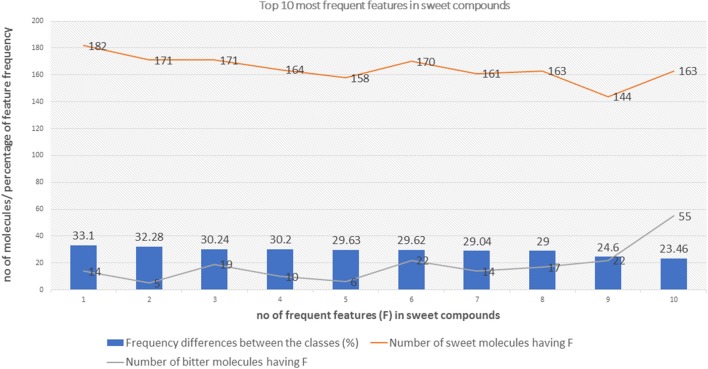
The distribution of top 10 most occuring frequent features in the sweet compounds and their relative occurences in the bitter class.

**Figure 2:**
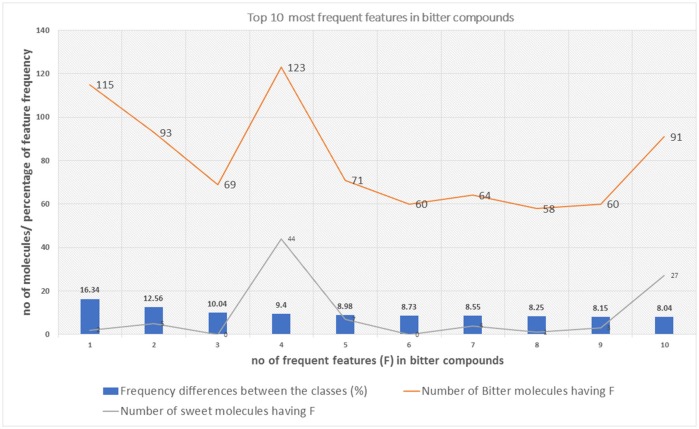
The distribution of top 10 most occuring frequent features in the bitter compounds and their relative occurences in the sweet class.

**Figure 3:**
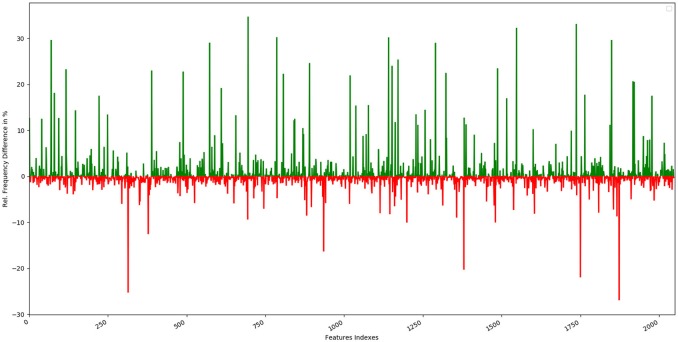
Graphical representation of the relative frequency distribution of each feature index in the sweet class (green) and bitter class (red) for Morgan fingerprints (2,048 bits).

**Figure 4:**
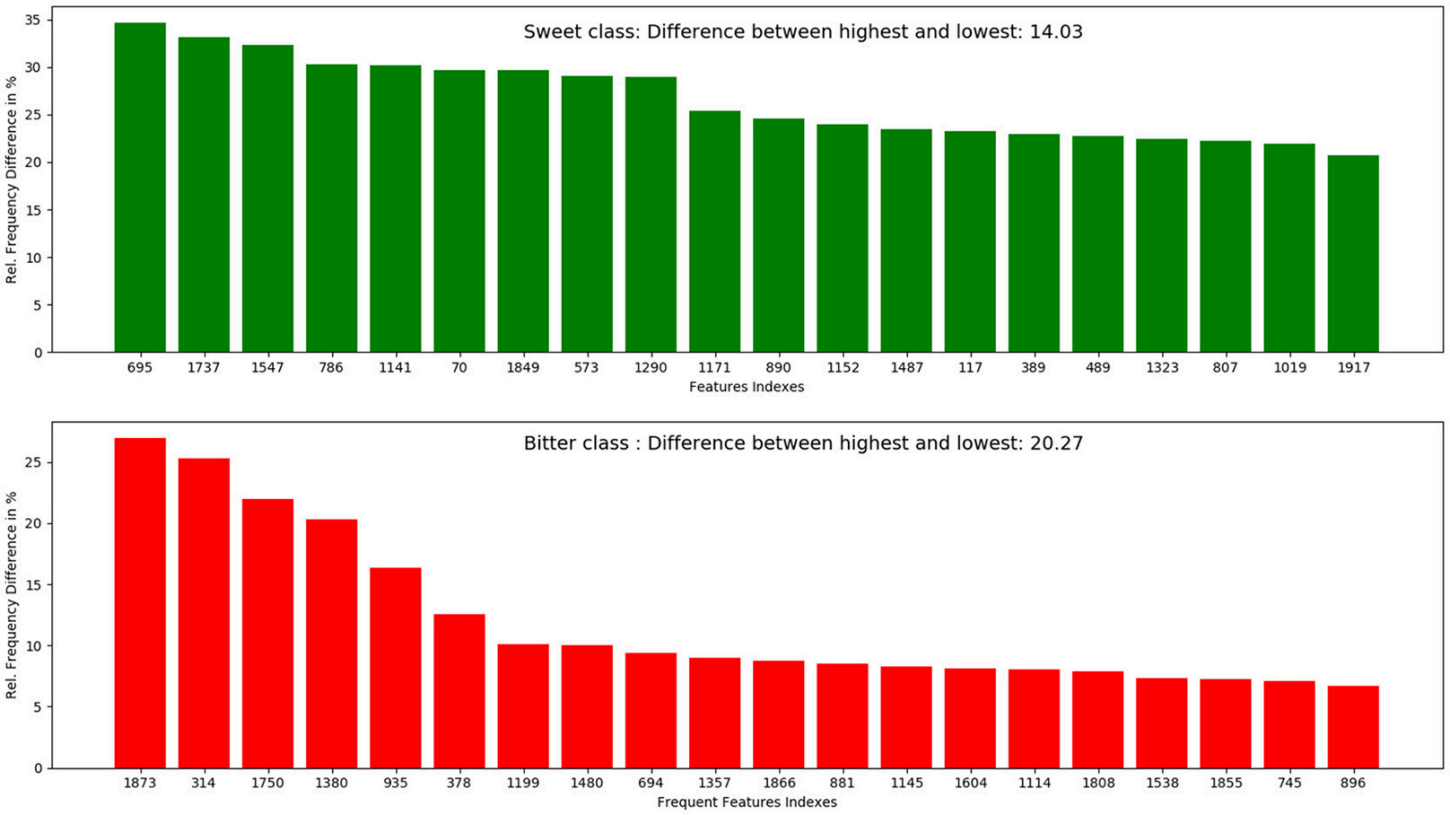
Top 20 most occurring features and their respective index position in both sweet and bitter molecules. It can be inferred from the figure that the top occurring features between sweet and bitter compounds used in this model are highly independent as individual index position in the fingerprints (bits set to 1) differs.

The top 10 most occurring features in respective classes and their relative frequency in each class are shown in (Figures 1, 2). The top 10 features of the sweet compounds tend to be more independent from the bitter compounds (Figure 1). On the other hand, the some of the sweet molecules seems to exhibit similar features when compared to bitter molecules (Figure 2). It is noticeable that the first three features of the bitter class are more dominant in the bitter molecules; remaining seven features are not strongly independent from the sweet class. The relationship between sweetness and bitterness of compounds are not uniformly linear, however it can be said that bitterness of some sweet compounds can increase or decrease in intensity as a function based on the presence of bitter-related features in them. From the prediction of the BitterSweetForest classifier and features assessment, it can be inferred that increases in bitterness of sweet-tasting compounds are bitter specific feature dependent. More details on the structures of the top features present in both the classes are provided in the Supplementary Figures 1, 2. Additionally, the relative frequency distribution of all 2,048 features in both classes was also computed (Figures 3, 4). It is observed that the indexes containing the frequent features in sweet and bitter compounds are different signifying that the some chemical features are class specific.

### Application of the model

Application of a model is as important as its development. The number of new chemicals discovered every year has been increasing over last few years with the development in synthetic chemistry research (Karaman, 2013; Bahia et al., 2017). With the increase of overall chemical space opens a platform for discoveries of new taste molecules. In this study, BitterSweetForest Classifier was applied on the three largest datasets. Firstly, on natural products, this was to predict and suggest some novel sweet/bitter molecules for the community. Secondly, on approved drug dataset, to understand if bitterness is an important quality for medicinal application. Similarly, to check which therapeutic class tends to be mostly bitter in the drug chemical space. Finally, on the oral toxicity dataset, to analyze if toxic compounds in general tend to be bitter in taste. In the following sections, each of the applications is described in detail. Furthermore, taking an example compound “aspartame” which is predicted as sweet with a confidence score of 0.94, using the KNIME workflow is provided in the Supplementary Material.

#### Prediction of sweet or bitter taste of the natural compounds

In order to predict the sweet or bitter taste of the natural compounds, 325,508 compounds from SuperNatural II Database (Banerjee et al., 2015) were extracted. The Morgan fingerprint of the compounds were created using Rdkit KNIME node (Berthold et al., 2008). BitterSweetForest classifier was applied to predict sweetness as well as the bitterness of the compounds. Almost 200,000 compounds were predicted to bitter and 25,916 compounds predicted to be sweet, with a confidence score of above 0.60 (where 1 means maximum). To further screen the compounds, we applied a threshold for confidence scores of 0.95, which resulted in 197 sweet predicted compounds and 3,865 compounds as bitter. The assessment of the applicability domain (AD) of the model was done using the similarity values between the natural product compounds and training set of the model. The pair-wise Tanimoto similarity was calculated using the Morgan fingerprints. The predicted compounds SNID (supernatural ID) and predicted class, confidence scores are provided in the Supplementary Material.

#### Prediction of sweet or bitter taste of approved drugs

**Figure 5:**
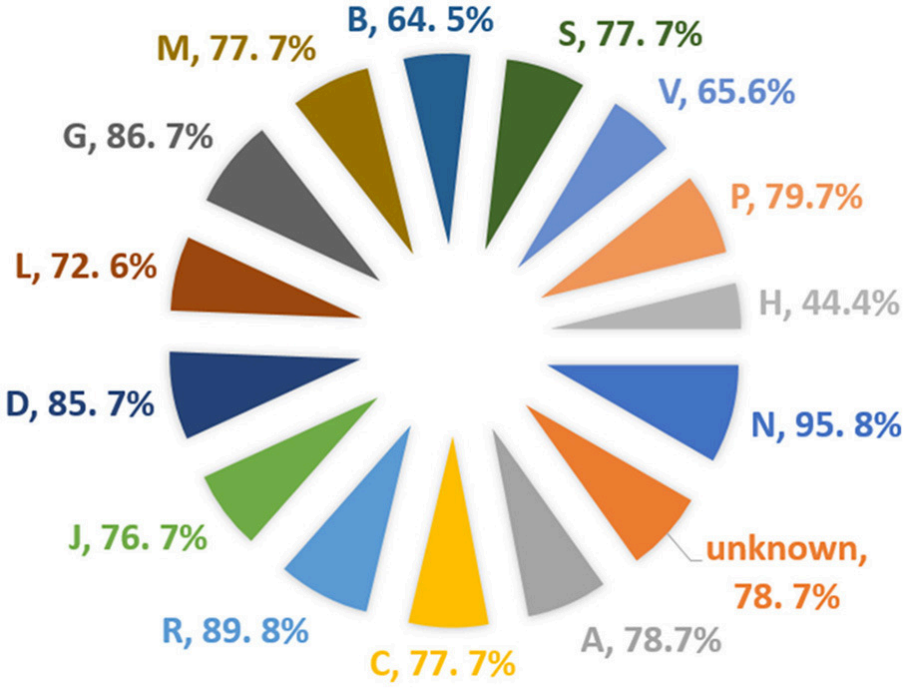
Percentage of approved drugs predicted as bitter and their corresponding Anatomical Therapeutic Chemical (ATC) class.

A total of 1,925 approved small molecule drugs were collected from DrugBank database (Wishart et al., 2017). After standardization and comparing with the dataset set of the model, almost 1,600 compounds were found to be within the applicability domain. Almost 77% of the total DrugBank (Wishart et al., 2017) approved dataset could be predicted using the BitterSweetForest classifier, with a confidence score of above 0.75. Out of the compounds predicted above the threshold of confidence score of 0.75, 98% of the drugs were predicted as bitter and 2% as sweet. This is interesting as bitterness is often connected to drugs when administrated orally. We further analyzed the Anatomical Therapeutic Chemical (ATC) (Nickel et al., 2014) classification of drugs (Figure 5); it was that 95% of the total drugs under the classification of Nervous System (N) were found to be bitter, not a single drug from this class was predicted as sweet above the threshold of 0.75. The second highest predicted class of drugs is Respiratory system (R) with a total of 89% of the drugs predicted as bitter. The third predicted class of drug is Genito urinary system and sex hormones (G) with 86.7% predicted as bitter. There was no ATC classification available for almost 231 drugs predicted as bitter. On the other hand, only 4.1% of the total drugs from ATC class various (V) were predicted as sweet with a confidence score of above 0.75. The DrugBank ID as well as the predicted taste class along with confidence scores for the prediction is provided in the Supplementary Material.

#### Prediction of sweet or bitter taste of the compounds present is oral toxicity dataset

Bitterness is directly proportional in relationship with our food intake: “bitter is bad”! Many poisons are known to taste bitter, a taste quality evoking a classic rejection response (Reed and Knaapila, 2010). These types of taste based rejection as well as acceptance of food are assumed to be inborn and unlearned, as it found to be apparent in human infants as well as non-human primates and in rodents (Rev, 2015).

The ProTox (Drwal et al., 2014), prediction server for oral toxicities of small molecules, was used in this study. The idea is “by evolution we know that substances which are toxic tend to be bitter.” However, the other way around may not be true, not all bitter substances are toxic; such as drugs are bitter compounds having health benefits. The classification of toxicity classes in Protox is based on the globally harmonized system of classification of labeling of chemicals. Toxicity class 1 is defined as fatal and toxicity class 6 as non-toxic. The prediction based on BitterSweetForest classifier, with a confidence score of 0.75 an above, for the compounds with their respective classes are given in Table 5. The prediction results suggest almost all the five toxic classes; have more bitter compounds and less sweet compounds, within the selected threshold. However, in toxicity class 1, which is considered as most toxic class, almost 75% of the total compounds were predicted as bitter compounds with a confidence score of 0.75 and above. Taken together, this suggests that acute oral toxic compounds tend to contain most of the features of bitter compounds and hence predicted as bitter in the study.

## Discussion

The classification performance for both sweet and bitter taste, by the proposed BitterSweetForest classifier achieved good performance both on cross-validation and on independent test set. Developing *in silico* approaches for the prediction of sweetness as well as bitterness of compounds has become a research focus in recent years (Di Pizio and Niv, 2014). Molecular fingerprints methods have been successfully applied in the computational prediction of bioactivities as well as toxicities, and have achieved good performance (Kurczab et al., 2011; Drwal et al., 2014, 2015; Lavecchia, 2015; Banerjee et al., 2016). Human testing of taste of chemicals is not only restricted from the regulation point of view, however is also limited due to ethical and toxicological issue. There is a greater need for the development of computational models for the prediction of tastes in general, to facilitate the taste chemical basic research. Recently, various computational classification models have been developed to predict the taste of chemical compounds, including QSTR based model (Rojas et al., 2017), Bitter X (Huang et al., 2016), BitterPredict (Dagan-Wiener et al., 2017). For comparison, the detailed classification accuracies, sensitivities, specificities as well as AUC values of these recently reported models were summarized in Tables 3, 4. Certainly, an intensive comparison of the results from the BitterSweetForest classifier with previously reported study is not completely feasible, because of the application of different sets of training sets used, number of molecular descriptors, and validation approach. However, a simple comparison could provide some basic information about the accuracy strength of the various prediction methods. Upon careful, comparison of the predictive performance of these computational models displayed in the Tables 3, 4, it was found that the prediction accuracies of the cross-validation and external validation of the BitterSweetForest classifier based on RF algorithm and Morgan fingerprints, established in this study was comparatively better than that of the other cited methods. The study suggests that the BitterSweetForest could give reasonable predictive performances in several evaluations parameters, and can be used for prediction of both sweet and bitter taste., The other advantage of this study is the analysis and identification of the most frequent features present in bitter and sweet compounds. The study reveals that some of the features of sweet compounds and bitter compounds are not completely independent from each other, and some features tends to be more class specific. However, if the presence of single feature in chemical compounds is alone responsible for a certain activity (i.e., is sweetness), this feature should be found in all the structures of sweet compounds and no bitter compounds should contain this feature and vice versa. The extension to this case increasing more number of features is not so simple. Let's say, if two features were essential for sweet taste, it would still be true that every sweet compound should contain both features, but up to half of the bitter compound might contain one of the two features and half the other feature. Although, such distinction between the sweet and bitter compounds cannot be as absolute as in the simple case of a single feature, each feature would be as twice as common in the sweet compounds as in the bitter compounds. Therefore, more analysis of these features with respective class of molecule will be needed in future. In general, it was observed that chemical environment of the features present in bitter compounds are more diverse. This could be because of the fact due to presence of multiple bitter receptors. On the other hand, sweet molecules have relatively very less diverse chemical environment in their features (Birch et al., 1981) as shown in Supplementary Figures 1, 2. Additionally, some of the sweet compounds as well as bitter compounds tend to exhibit features that are common between them.

**Table 3:** Comparison with top two methods predicting sweet taste of molecules.

**Methods**	**Set**	**Positive**	**Negative**	**NER (%)**	**Sensitivity**	**Specificity**
*K*NN (2016)	Training	–	–	90.00	–	–
	Test	–	–	0.92	–	–
QSTR (2017)	Training	327	161	83.00	0.77	0.89
	Test	108	53	85.0	0.79	0.91
BitterSweet Forest	Training	416	548	93.50	0.97	0.90
	Test	102	139	94.00	0.97	0.91

* NER, Ratio of correctly classified molecules to the total number of molecules.

**Table 4:** Comparison with top two methods predicting bitter taste of molecules.

**Methods**	**Set**	**Positives**	**Negatives**	**Accuracy (%)**	**Sensitivity**	**Specificity**	**AUC**
Bitter X (2016)	Training	431	431	88.00	–	–	–
	Test	108	108	91.00	0.90	0.91	0.94
BitterPredict (2017)	Training	484	1,343	93.00	0.91	0.94	–
	Test	207	574	83.00	0.77	0.86	–
BitterSweet Forest	Training	545	416	95.00	0.97	0.90	0.97
	Test	139	102	96.69	0.97	0.91	0.98

**Table 5:** BitterSweetForest prediction of the oral toxicity compounds.

**Toxicity class**	**Lethal dose (LD_50_ = X) in mg/kg**	**Total number of molecules per class**	**Predicted bitter molecules**	**Predicted sweet molecules**
1 (fatal)	X ≤ 5	510	384	0
2 (fatal)	5 < X ≤ 50	1,779	1,392	5
3 (toxic)	50 < X ≤ 300	6,918	5,579	3
4 (harmful)	300 < X ≤ 2,000	21,884	17,340	24
5 (may be harmful)	2,000 < X ≤ 5,000	6,740	5,413	26

Furthermore, the BitterSweetForest was applied for the prediction of natural compounds from the SuperNatural II (Banerjee et al., 2015) database containing 325,508 natural products. The classifier was further applied to predict the approved drug dataset from DrugBank (Wishart et al., 2017) database and the oral toxicity dataset from the Protox (Drwal et al., 2014) dataset. It is generally observed that most of the chemical from all the three sets (SuperNaturall II; DrugBank as well as ProTox) tends to exhibit more percentage of bitter features with a confidence score of 0.75 and above. It could also be the fact that considering such a strict threshold, most of the predicted sweet compounds on the three sets did not qualify on the final result set. The threshold of 0.75 was considered mostly to reduce the noise and false prediction that could arise to due to applicability domain. The applicability domain of the datasets was accessed by computing pair-wise similarity with the training set molecules, using Morgan fingerprint. It is observed in the study, that most of the highly predicted drugs as bitter compounds belong to the ATC class N, R, D. The toxic compounds were predicted mostly as bitter compounds by the BitterSweetForest classifier.

Overall, the simplicity and consistent robust performance of the BitterSweetForest classification model will make it a helpful tool in assisting scientists to propose sweet as well as bitter compounds either by synthesis or by virtual screening of very large chemical libraries. The predictive performance of the model is comparatively and significantly better than the models reported previously. The cost associated in training the model was very low, adding further to the usability of the BitterSweetForest classifier. Most of the previously reported classification models either are available only as web-server or built on descriptors based on commercial software, which can limit the use by the community who would like to patent their novel structures and therefore, may not like to release their structure on public webservers. Hence, BitterSweetForest model was constructed using an open source KNIME as a simple workflow, which will enable the community to use the model locally. Only basic computational skills are required for using this workflow. In the future, we would like to relate the mechanism of action between the receptors (bitter and sweet) and their respective features. Also, it will be beneficial to analyze the presence of toxic fragments in both bitter and sweet compounds. It is also planned to compute the features dependence and their activity profiles for both bitter and sweet compounds from the discriminative chemical features identified in this study.

## Conclusions

In this study, a binary combined prediction model for bitter and sweet taste of small compounds is constructed. The constructed model yielded accuracy of 95% and AUC of 0.98 in cross-validation. In independent test, BitterSweetForest achieved accuracy of 96% and AUC of 0.98 for bitter and sweet taste prediction. The top frequent occurring features were analyzed for both the classes. Additionally, the feature distribution for each bit position from Morgan fingerprint consisting of 2,048 bits, in respective classes was computed. BitterSweetForest to best of our knowledge is the first freely available KNIME based workflow platform for the community and will be useful resource for basic taste chemistry related research as well as new sweet and bitter tasting compounds discoveries in the industry. Furthermore, the prediction model was applied to predict both sweet and bitter tasting compounds from natural products (Banerjee et al., 2015). The approved drug set from the DrugBank (Wishart et al., 2017) dataset was also predicted and it was found that most of the drugs tend to taste bitter. The prediction on oral toxicity dataset from ProTox (Drwal et al., 2014), reveals that most toxic compounds are bitter in taste. From this study, it could be inferred that drugs as well as toxic compounds do exhibit bitter specific features. This will be indeed interesting to explore, if quantification of bitterness can be used to define both therapeutic as well as toxic endpoints in a chemical structure.

The KNIME workflow for BitterSweetForest classifier will be made available for download via the link http://bioinformatics.charite.de/sweet/.

## Author contributions

PB and RP conceived the study. PB designed and performed the study. PB wrote the manuscript. PB and RP proofread the manuscript. All authors read and approved the final manuscript.

### Conflict of interest statement

The authors declare that the research was conducted in the absence of any commercial or financial relationships that could be construed as a potential conflict of interest.

## Abbreviations

RF: Random Forest
ROC: receiver operating characteristic
AUC: area under the curve
ATC: anatomical therapeutic chemical
R: Respiratory system
G: Genito urinary system and sex hormones
D: Dermatologicals
P: Antiparasitic products, insecticides and repellents
A: Alimentary tract and metabolism
C: Cardiovascular System
M: Musculo-skeletal system
S: Sensory Organs
J: Anti-infectives for systemic use
L: Antineoplastic and immunomodulating agents
V: Various
B: Blood and blood forming organs
H: Systemic hormonal, preparations, excluding sex hormones and insulins, unknown- for ATC classification is not available.
