# Supplementary figures and images for: BitterSweetForest: A Random Forest Based Binary Classifier to Predict Bitterness and Sweetness of Chemical Compounds
Source: Front Chem. 2018 Apr 11;6:93. doi: 10.3389/fchem.2018.00093 (PMC5905275; doi:10.3389/fchem.2018.00093)

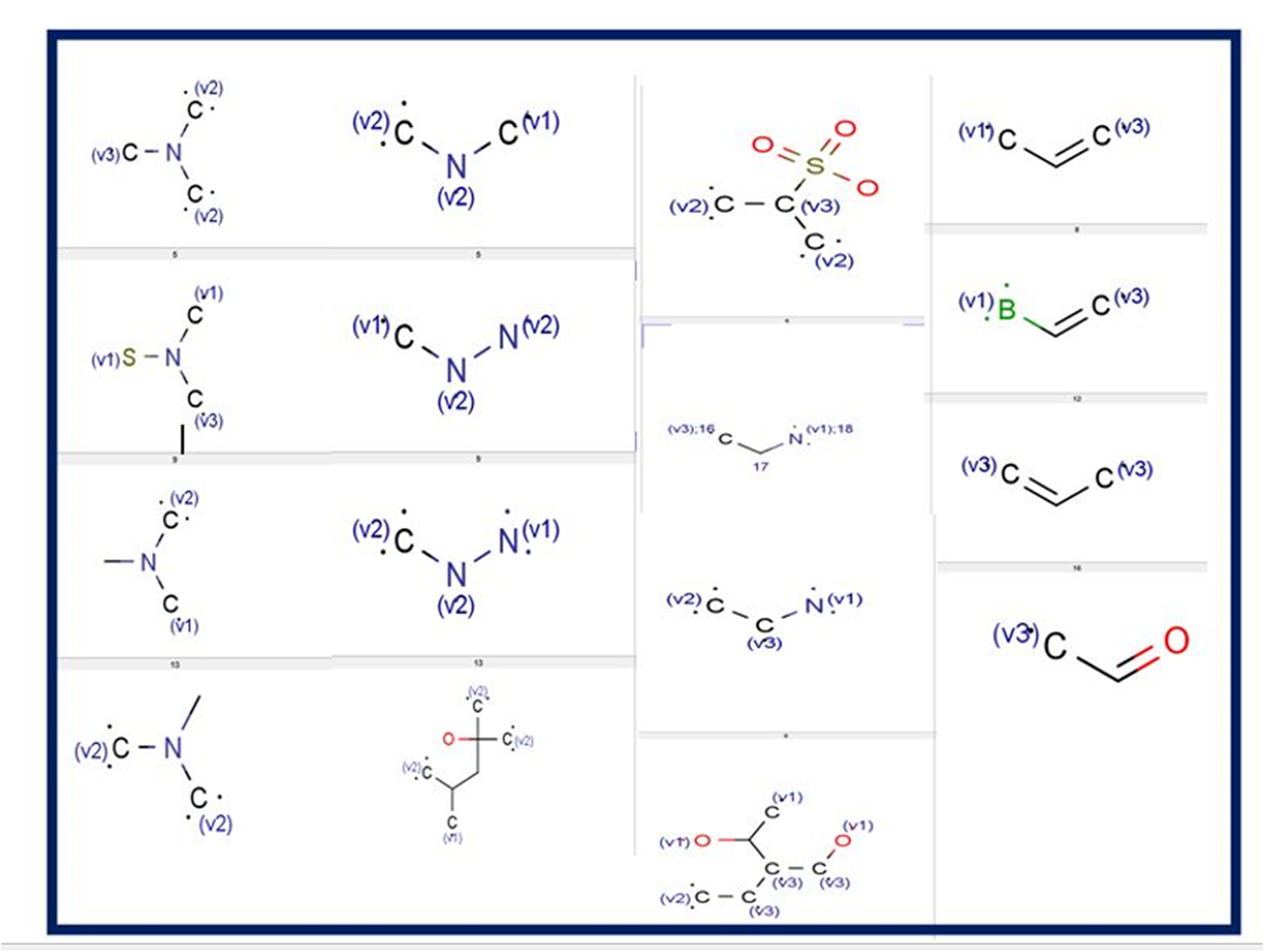

Supplement: Supplementary file 1 [file Image1.TIF]

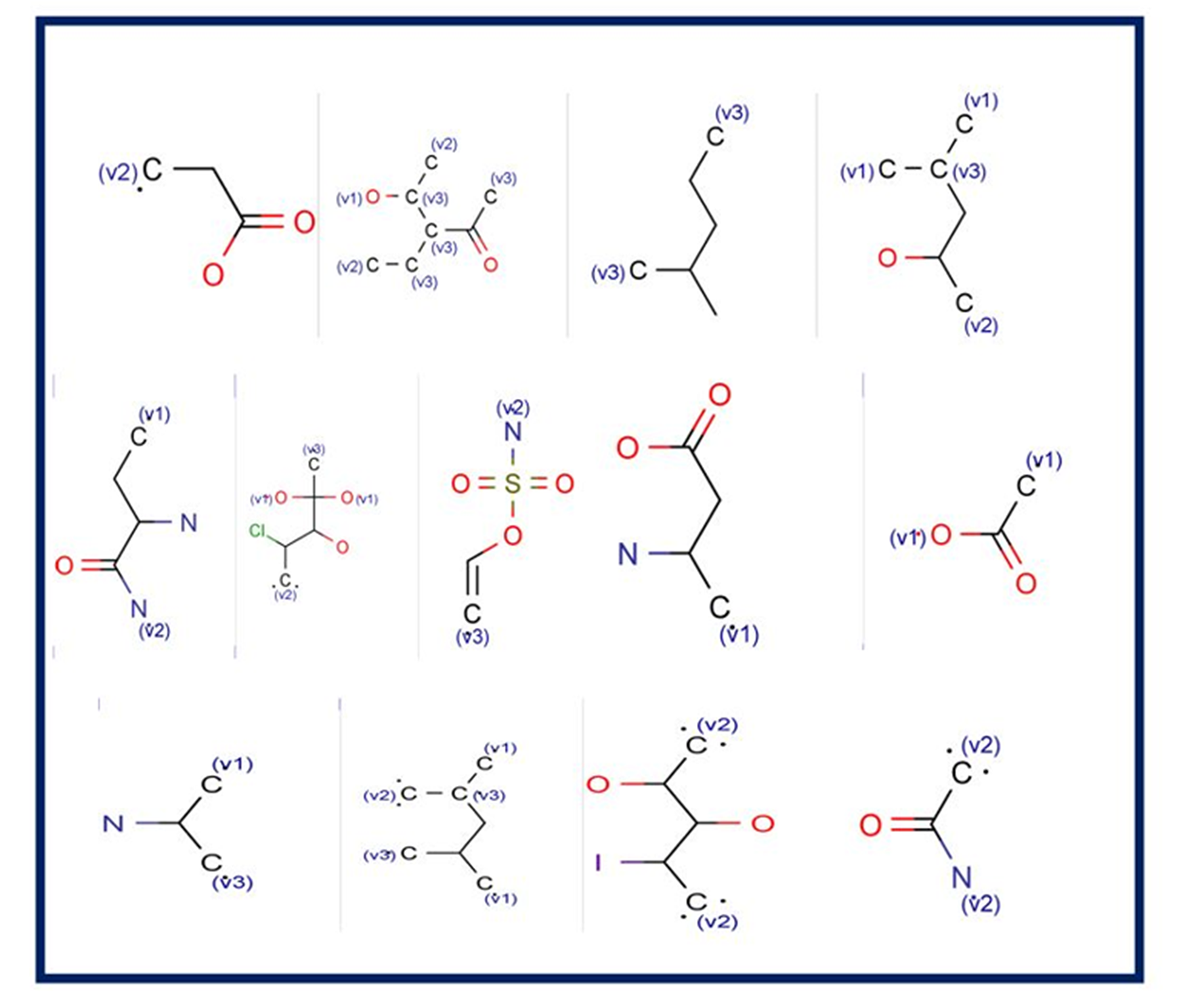

Supplement: Supplementary file 2 [file Image2.TIF]

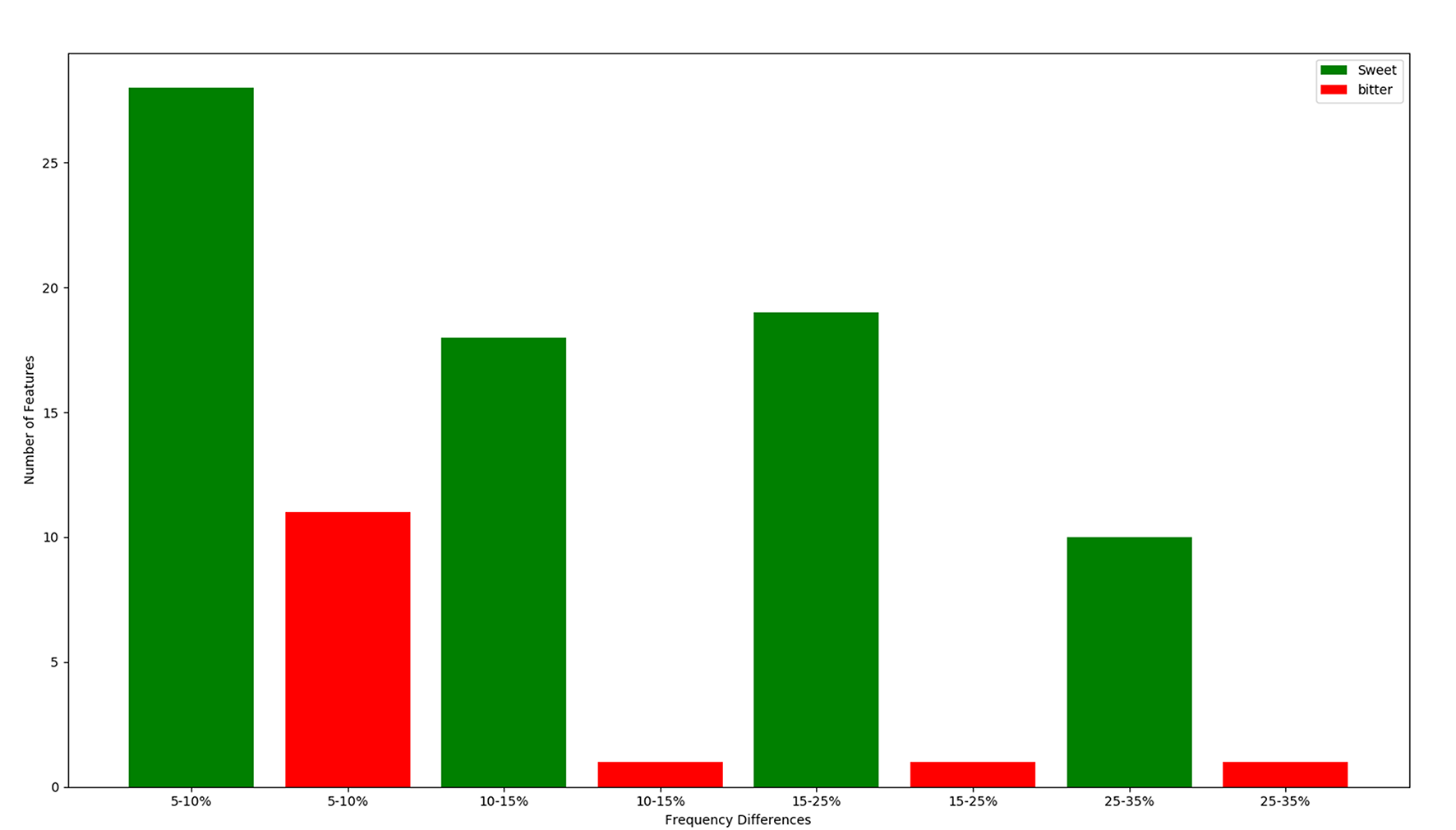

Supplement: Supplementary file 3 [file Image3.TIFF]

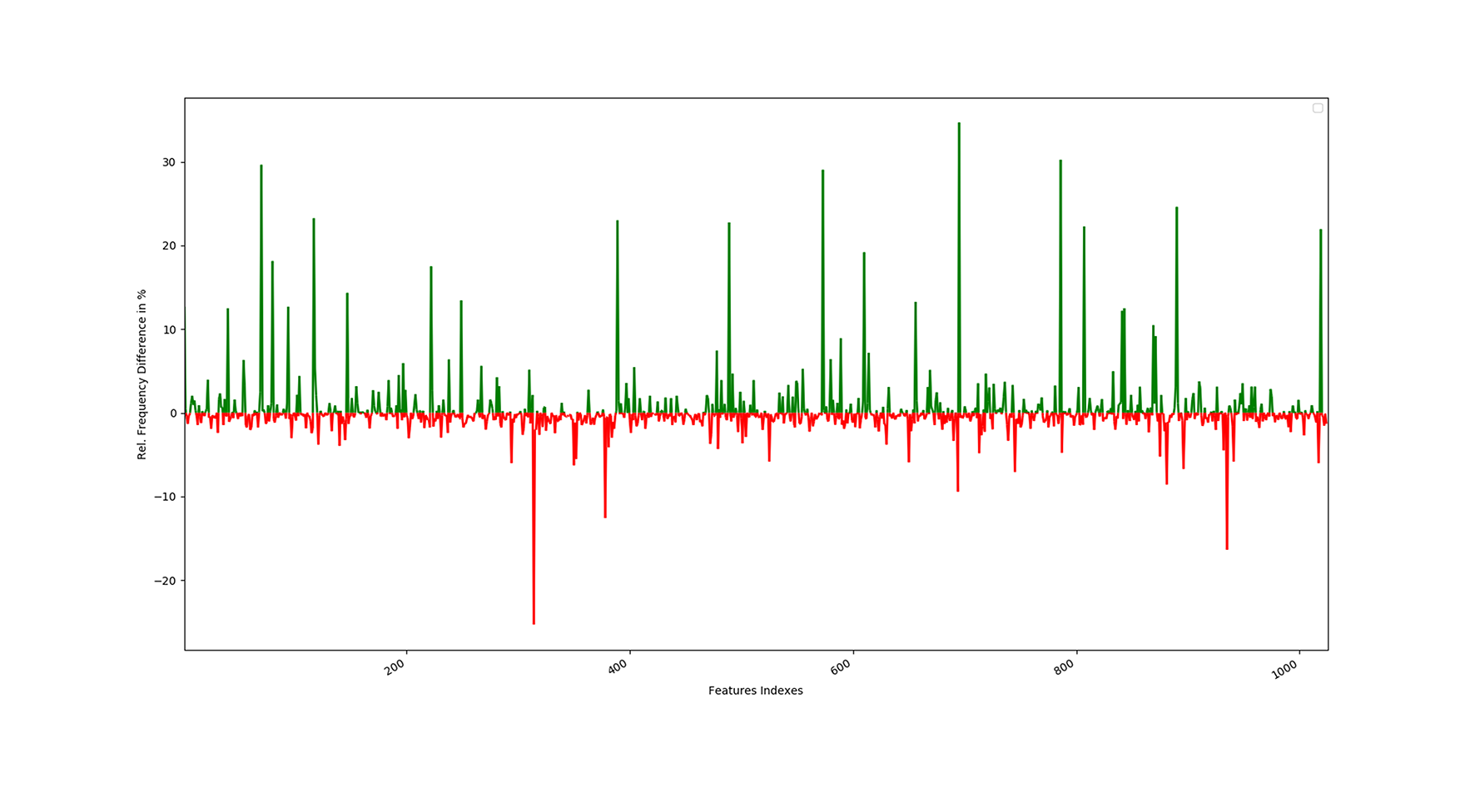

Supplement: Supplementary file 4 [file Image4.TIF]

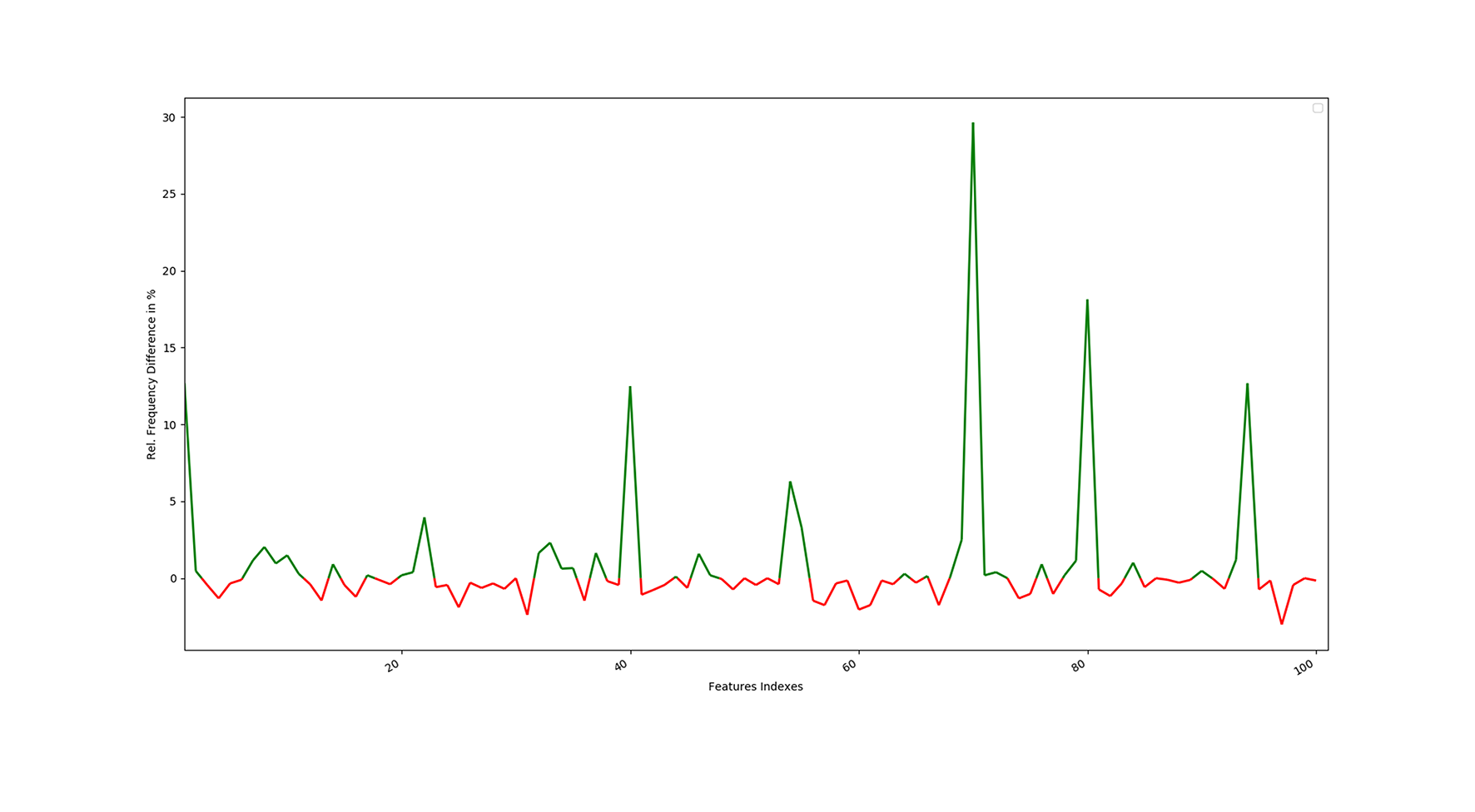

Supplement: Supplementary file 5 [file Image5.TIF]

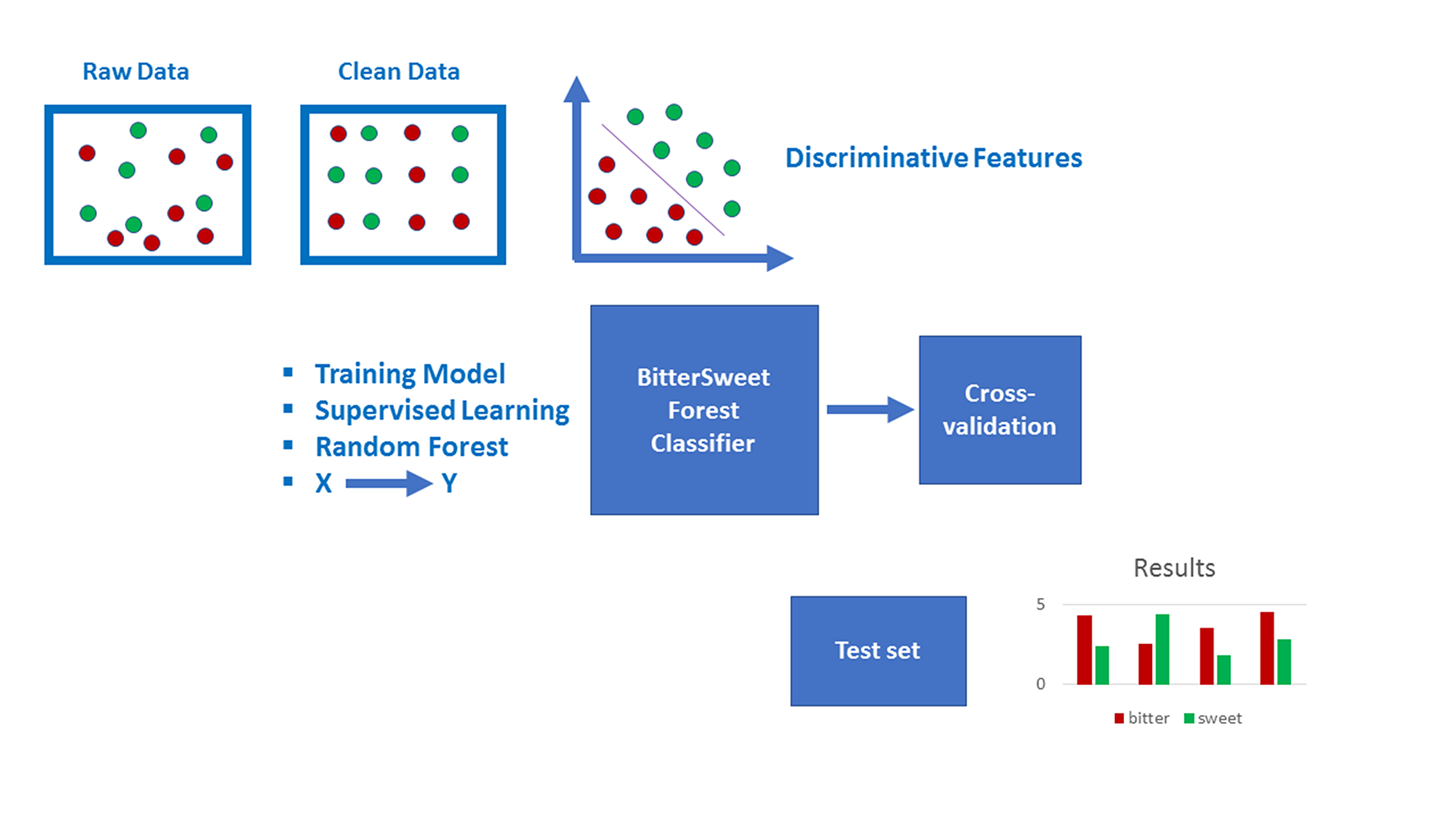

Supplement: Supplementary file 6 [file Image6.TIF]

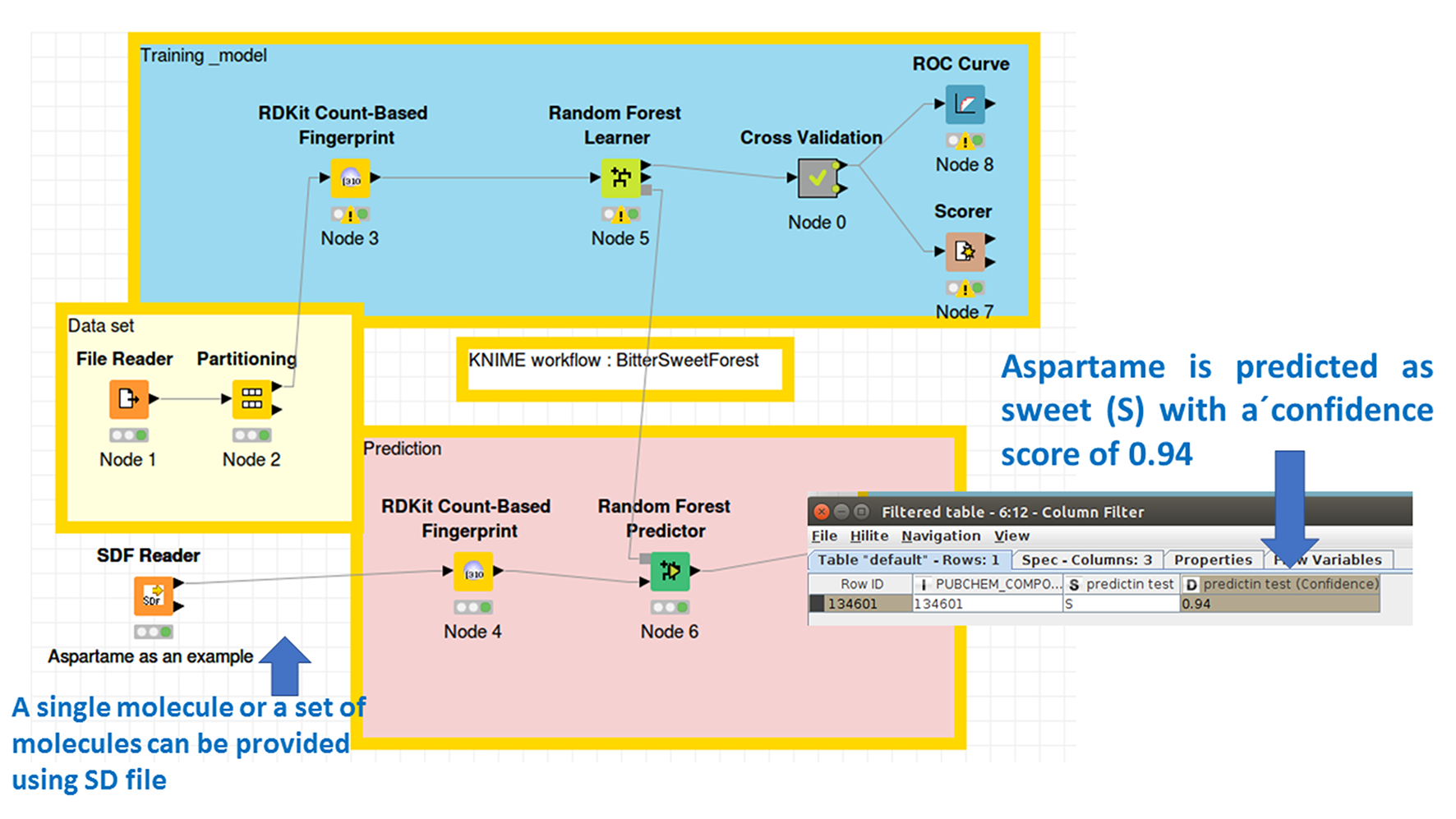

Supplement: Supplementary file 7 [file Image7.TIF]
