# Supplementary material for: BitterSweetForest: A Random Forest Based Binary Classifier to Predict Bitterness and Sweetness of Chemical Compounds
Source: Front Chem. 2018 Apr 11;6:93. doi: 10.3389/fchem.2018.00093 (PMC5905275; doi:10.3389/fchem.2018.00093)
Supplement: Supplementary file 8 [file Table1.docx]

Supplementary Material

BitterSweetForest: A random forest based binary classifier to predict bitterness and sweetness of chemical compounds

Priyanka Banerjee*, Robert Preissner

*** Correspondence:** Corresponding Author: Robert.preissner@charite.de

# Supplementary Figures and Tables

Figure legends:

Supplementary Figure 1: Some of the examples of top occurring features in bitter compounds

Supplementary Figure 2: Some of the examples of top occurring features in sweet compounds

Supplementary Figure 3: The top occurring features in the entire dataset and their relative frequency in the respective sweet and bitter class

Supplementary Figure 4: Examples of relative feature distribution in sweet (green) and bitter (red) for first 100 bits, as computed using Morgan fingerprint.

Supplementary Figure 5: Examples of relative feature distribution in sweet (green) and bitter (red) for first 1024 bits respectively, as computed using Morgan fingerprint.

Supplementary Figure 6: The schematic representation of the workflow behind the BitterSweetForest classifier

Supplementary Figure 7: Diagrammatic representation of the KNIME workflow

Supplementary Table 1: Results of BitterSweetForest Classifier using different fingerprints

| **Fingerprints** | **Cross-validation**  **accuracy** | **External validation accuracy** | **Cross-validation AUC** | **External**  **AUC** |
| --- | --- | --- | --- | --- |
| Morgan | 95.00 | 96.69 | 0.88 | 0.92 |
| Morgan-Feat | 94.35 | 94.16 | 0.98 | 0.97 |
| Atom pair | 94.40 | 95.00 | 0.98 | 0.96 |
| Torsion | 93.34 | 93.33 | 0.97 | 0.97 |

**
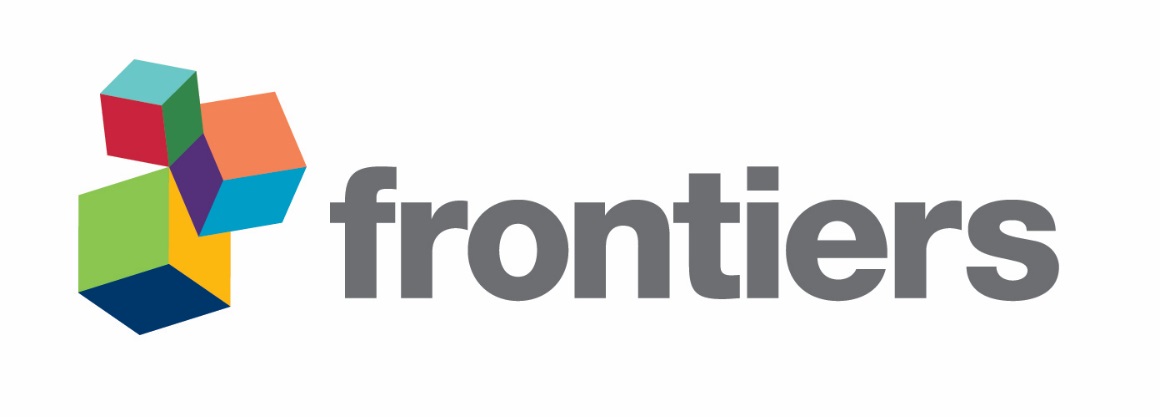
**
